# DeepFruits: A Fruit Detection System Using Deep Neural Networks

**DOI:** 10.3390/s16081222

**Published:** 2016-08-03

**Authors:** Inkyu Sa, Zongyuan Ge, Feras Dayoub, Ben Upcroft, Tristan Perez, Chris McCool

**Affiliations:** Science and Engineering Faculty, Queensland University of Technology, Brisbane 4000, Australia; gzy555555@gmail.com (Z.G.); feras.dayoub@qut.edu.au (F.D.); ben.upcroft@qut.edu.au (B.U.); tristan.perez@qut.edu.au (T.P.); c.mccool@qut.edu.au (C.M.)

**Keywords:** visual fruit detection, deep convolutional neural network, multi-modal, rapid training, real-time performance, harvesting robots, horticulture, agricultural robotics

## Abstract

This paper presents a novel approach to fruit detection using deep convolutional neural networks. The aim is to build an accurate, fast and reliable fruit detection system, which is a vital element of an autonomous agricultural robotic platform; it is a key element for fruit yield estimation and automated harvesting. Recent work in deep neural networks has led to the development of a state-of-the-art object detector termed Faster Region-based CNN (Faster R-CNN). We adapt this model, through transfer learning, for the task of fruit detection using imagery obtained from two modalities: colour (RGB) and Near-Infrared (NIR). Early and late fusion methods are explored for combining the multi-modal (RGB and NIR) information. This leads to a novel multi-modal Faster R-CNN model, which achieves state-of-the-art results compared to prior work with the F1 score, which takes into account both precision and recall performances improving from 0.807 to 0.838 for the detection of sweet pepper. In addition to improved accuracy, this approach is also much quicker to deploy for new fruits, as it requires bounding box annotation rather than pixel-level annotation (annotating bounding boxes is approximately an order of magnitude quicker to perform). The model is retrained to perform the detection of seven fruits, with the entire process taking four hours to annotate and train the new model per fruit.

## 1. Introduction

According to [1], sourcing skilled farm labour in the agriculture industry (especially horticulture) is one of the most cost-demanding factors in that industry. This is due to the rising values of supplies, such as power, water irrigation, agrochemicals, and so on. This is driving farm enterprises and horticultural industry to be under pressure with small profit margins. Under these challenges, food production still needs to meet the growing demands of an ever-growing world population, and this casts a critical problem to come.

**Figure 1:**
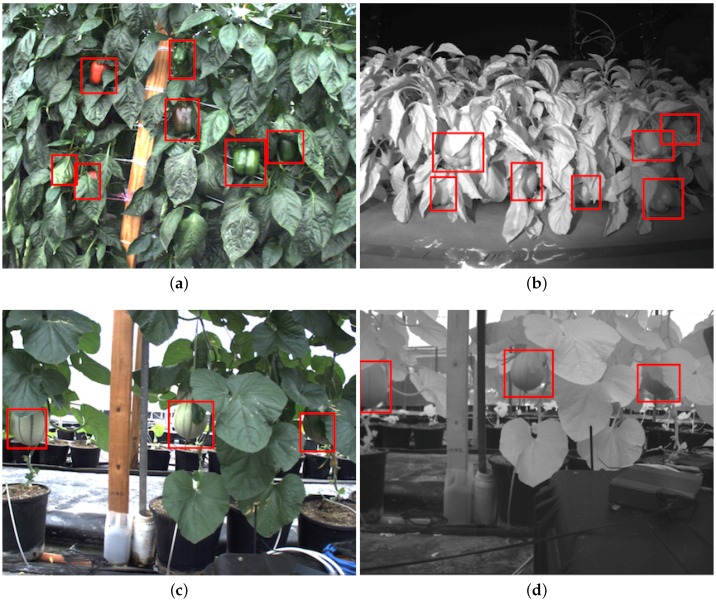
Example images of the detection for two fruits. (**a**) and (**b**) show a colour (RGB) and a Near-Infrared (NIR) image of sweet pepper detection denoted as red bounding boxes respectively. (**c**) and (**d**) are the detection of rock melon.

Robotic harvesting can provide a potential solution to this problem by reducing the costs of labour (longer endurance and high repeatability) and increasing fruit quality. For these reasons, there has been growing interest in the use of agricultural robots for harvesting fruit and vegetables over the past three decades [2,3]. The development of such platforms includes numerous challenging tasks, such as manipulation and picking. However, the development of an accurate fruit detection system is a crucial step toward fully-automated harvesting robots, as this is the front-end perception system before subsequent manipulation and grasping systems; if fruit is not detected or seen, it cannot be picked. This step is challenging due to various factors, among which are illumination variation, occlusions, as well as the cases when the fruit exhibits a similar visual appearance to the background, as shown in Figure 1. To overcome these, a well-generalised model that is invariant and robust to brightness and viewpoint changes and highly discriminative feature representations are required.

In this work, we present a rapid training (about 2 h on a K40 GPU) and real-time fruit detection system based on Deep Convolutional Neural Networks (DCNN) that can generalise well to various tasks with pre-trained parameters. It can be also easily adapted to different types of fruits with a minimum number of training images. In addition, we introduce approaches that combine multiple modalities of information (colour and near-infrared images) with early and late fusion. For the evaluation, we demonstrate both quantitative and qualitative results compared to previous work [4]. The contributions of this paper are therefore:

- Developing a high-performance fruit detection system that can be rapidly trained with a small number of images using a DCNN that has been pre-trained on a large dataset, such as ImageNet [5].
- Proposing multi-modal fusion approaches that combine information from colour (RGB) and Near-Infrared (NIR) images, leading to state-of-the-art detection performance.
- Returning our findings to the community through open datasets and tutorial documentation [6].

To the best of our knowledge, this is the first attempt to fuse RGB and NIR multi-modal images within a DCNN framework for fruit detection. We use standard evaluation metrics, precision-recall curves and the F1 score [7] (i.e., the harmonic mean of precision and recall), to perform extensive evaluations using data collected from three commercial sites acquired during day and night. This dataset, along with the annotated ground truth imagery and labelling tool will be distributed upon the publication of this work to encourage further research use in the relevant area.

The remainder of the paper consists of the following. Section 2 introduces related work and the background. Section 3 presents the descriptive comparisons between our previous works using the Conditional Random Field (CRF) with hand-crafted features and the proposed approach using Faster Region-based Convolutional Neural Network (R-CNN) for fruit detection. Multi-modal fusion schemes are also addressed in this section. We demonstrate the experimental results in Section 4. Conclusions are drawn in Section 6.

## 2. Related Work/Background

Although many researchers have tackled the problem of fruit detection, such as the works presented in [8,9,10,11,12,13], the problem of creating a fast and reliable fruit detection system persists, as found in the survey by [14]. This is due to high variation in the appearance of the fruits in field settings, including colour, shape, size, texture and reflectance properties. Furthermore, in the majority of these settings, the fruits are partially abstracted and subject to continually-changing illumination and shadow conditions.

Various works presented in the literature address the problem of fruit detection as an image segmentation problem (i.e., fruit vs. background). Wang et al. [11] examined the issue of apple detection for yield prediction. They developed a system that detected apples based on their colour and distinctive specular reflection pattern. Further information, such as the average size of apples, was used to either remove erroneous detections or to split regions that could contain multiple apples. Another heuristic employed was to accept as detections only those regions that were mostly round. Bac et al. [12] proposed a segmentation approach for sweet peppers. They used a six band multi-spectral camera and used a range of features, including the raw multispectral data, normalised difference indices, as well as entropy-based texture features. Experiments in a highly controlled glasshouse environment showed that this approach produced reasonably accurate segmentation results. However, the authors noted that it was not accurate enough to build a reliable obstacle map.

Hung et al. [13] proposed the use of conditional random fields for almond segmentation. They proposed a five-class segmentation approach, which learned features using a Sparse Autoencoder (SAE). These features were then used within a CRF framework and was shown to outperform previous work. They achieved impressive segmentation performance, but did not perform object detection. Furthermore, they noted that occlusion presented a major challenge. Intuitively, such an approach is only able to cope with low levels of occlusion.

More recently, Yamamoto et al. [10] performed tomato detection by first performing colour-based segmentation. Then, colour and shape features were used to train a Classifier and Regression Trees (CART) classifier. This produced a segmentation map and grouped connected pixels into regions. Each region was declared to be a detection and to reduce the number of false alarms. They trained a non-fruit classifier using a random forest in controlled glasshouse environments.

In all of the above-mentioned works, a pixel-level segmentation approach for object detection has been adopted, and most of these works have examined fruit detection predominantly for yield estimation [8,11]. The limited studies that have conducted accurate fruit detection have done so for fruits in controlled glasshouse environments. As such, the issue of fruit detection in highly challenging conditions remains unsolved. This is due to the high variability in the appearance of the target objects in the agricultural settings, which meant that the classic methods of sliding window approaches, although showing good performance when tested on datasets of selected images [15], cannot handle the variability in scale and appearance of the target objects when deployed in real farm settings.

Recently, deep neural networks have made considerable progress in object classification and detection [5,16,17]. The state-of-the-art detection framework on PASCAL-VOC [18] consists of two stages. The first stage of the pipeline applies a region proposal method, such as selective search [19] and edgebox [20] to extract regions of interest from an image and then feed them to a deep neural network for classification. Although it has high recall performance, this pipeline is computationally expensive, which prevents it from being used in real time for a robotic application. Region Proposal Networks (RPNs) [21,22,23] solve this problem by combining a classification deep convolutional network with the object proposal network, so the system can simultaneously predict object bounds and classify them at each position, the parameters of the two networks are shared, which results in a much faster performance, making it suitable for robotic applications.

In real outdoor farm settings, a single sensor modality can rarely provide the needed information to detect the target fruits under a wide range of variations in illumination, partial occlusions and different appearances. This makes a great case for the use of multi-modal fruit detection systems because varying types of sensors can provide complementary information regarding different aspects of the fruits. Deep neural networks have already shown great promise when used for multi-modal systems in domains outside agricultural automation, such as in [24], where audio/video has been used very successfully, and in [25,26], where image/depth demonstrate a better performance compared to the utilisation of each modality alone. This work follows the same approach and demonstrates the use of a multi-modal region-based fruit detection system and how it outperforms pixel-level segmentation systems, as we show in the following sections.

## 3. Methodologies

Fruit segmentation is an essential step in order to distinguish the fruits from the background (leaves and stems). This task is challenging due to variation in fruit colour and illumination, as well as high levels of occlusion.

In this section, we present the state-of-the-art fruit detection system [4], which performs pixel-wise segmentation, against which we compare. We then describe the DCNN approach, Faster R-CNN, which forms the basis of our proposed method. The details behind adapting this model for fruit detection are then given, followed by a description of the fusion methods we propose for this DCNN architecture.

### 3.1. Fruit Detection Using a Conditional Random Field

In prior work [4], we demonstrated that using a CRF [27] to model colour and visual texture features from multi-spectral images led to the impressive performance for sweet pepper segmentation. The multi-spectral images contain both colour (RGB) and Near-Infrared (NIR) information. The CRF uses both colour and texture features. The colour features are constructed by directly converting the RGB values to the HSV colour space. Visual texture features are extracted from the NIR channel. NIR images are used to calculate texture features, as they were found to be more consistent than the colour imagery. Three sets of visual texture features are used: (i) Sparse Autoencoder (SAE) features [13]; (ii) Local Binary Pattern (LBP) [28]; and (iii) a Histogram of Gradients (HoG) [29]. Each feature captures a different property, such as the distribution of the local gradient, edges and texture, respectively. It appears that the LBP feature can capture information, such as the smooth surface of sweet peppers, and provides an efficient method for encoding visual texture.

**Figure 2:**
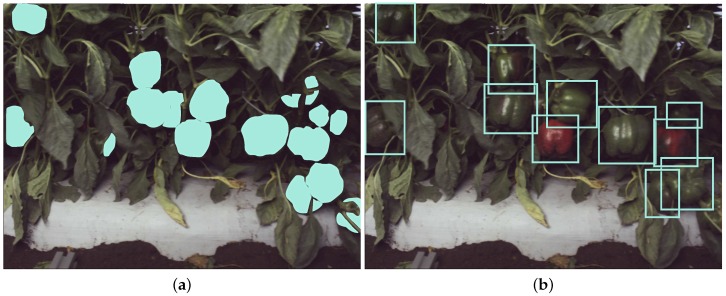
Pixel-wise (**a**) and bounding box (**b**) image annotation.

Although the CRF-based approach yields impressive results, there are two key challenges: ground truthing (i.e., pixel-wise image annotation) for training, and model evaluation requires labour-intensive work, as shown in Figure 2. Pixel-wise annotations took an order of magnitude more time to produce than bounding box annotations (in our experiments per-pixel segmentation of 5 images took approximately 470 s, whereas bounding box annotations took approximately 43 s). The slow processing time (∼1.5 s/frame only using LBP feature) of the current MATLAB implementation is also a bottleneck for robotic application, which usually requires a closed-loop control. We present quantitative comparisons to our new proposed method in Section 4.2.

### 3.2. Fruit Detection Using Faster R-CNN

Despite the recent progress being made using deep convolutional neural networks on large-scale image classification and detection [5], accurate object detection still remains a challenging problem in the computer vision and machine learning fields. This task requires not only detecting which objects are in a scene, but also where they are located. Accurate region proposal algorithms thus play significant roles in the object detection task.

There are recent works, such selective search [19], which merges super pixels based on low-level features, and EdgeBoxes [20], making use of edge information to generate region proposals. However, these methods require as much running time as the detection to hypothesise object locations. Faster R-CNN [21] was proposed to overcome this challenge by introducing the Region Proposal Network (RPN), which shares convolutional features with the classification network, and two networks are concatenated as one network that can be trained and tested through an end-to-end process. By doing that, the running time for region proposal generation takes around 10 ms, and this framework can maintain a 5 fps detection rate and outperform the state-of-the-art object detection accuracy using very deep models [30].

The Faster R-CNN work of [21] uses colour (RGB) images to perform general object detection. It consists of two parts: (i) region proposal; and (ii) a region classifier. The region proposal step produces a set of $N_{P}$ proposed regions (bounding boxes) where the object(s) of interest could reside within the image. The region classifier step then determines if the region belongs to an object class of interest; the classifier could be a 2-class or *N*-class classifier. To train the Faster R-CNN for our task, we perform fine-tuning [31]. This requires labelled (annotated) bounding box information for each of the classes to be trained. An example of the bounding boxes required is given in Figure 2.

**Figure 3:**
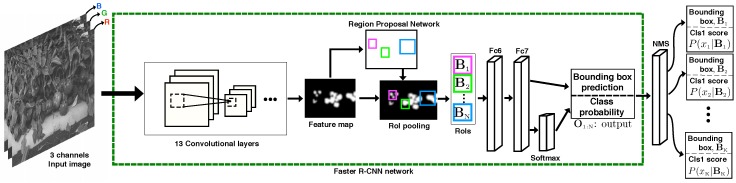
Illustration of test time the Faster Region-based Convolutional Neural Network (R-CNN). There are 13 convolutional and 2 fully-connected (Fc6 and Fc7) and one softmax classifier layers. *N* denotes the number of proposals and is set as 300. $O_{1:N}$ is the output that contains *N* bounding boxes and their scores. Non-Maximum Suppression (NMS) with a threshold of 0.3 removes duplicate predictions. $B_{K}$ is a bounding box of the *K*-th detection that is a 4 × 1 vector containing the coordinates of top-left and bottom right points. xK is a scalar representing an object being detected.

Figure 3 illustrates the test-time detection pipe line. First, regions of interest are generated from the input image, and these are fed into subsequent convolutional layers. In this paper, we use the VGG-16 model that has 13 convolutional layers. The RPN produces region proposals using the previously-generated feature map. These proposals highlight the regions that are highly probable to contain an object. Fully-connected layers and the softmax classifier yield n bounding boxes, $B_{n}$ and their corresponding probability scores of each class, $P(x_{n}|B_{n})$.

**Figure 4:**
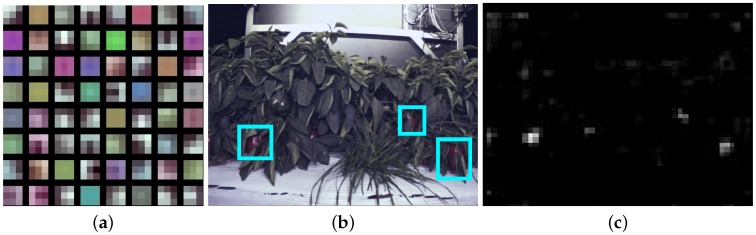
(**a**) The 3 × 3 (pixels) Conv164 filters of the RGB network from VGG, (**b**) The input data and (**c**) One of the feature activations from the conv5 layer. The cyan boxes in (b) are manually labelled in the data input layer to highlight the corresponding fruits of the feature map.

Although it is usually challenging and out of the scope of this paper to prove why the use of deep convolutional neural network works well for the object detection task, we present some level of visual proofs that the neural networks can capture significant features discriminatively. Figure 4a is the visualisation of the first convolutional layer of the colour VGG-16 network. This model is designed to use of 3 × 3 convolutional kernels (mask) and a 2 × 2 pooling mask from the beginning to the end of 13 convolutional nets. It can be observed that filters have reddish and greenish colours that correspond to red and green sweet peppers. Other filters represent edge filters in varying orientations. Figure 4b shows the input data layer and one of feature maps from conv5 layer Figure 4c. It can be seen that the regions for sweet red peppers (cyan boxes) are strongly activated, and this information is highly useful for RPN and further classification process.

We also perform further investigation for visual proofs by visualising a high-dimensional feature space. It was shown that output from the fully-connected layer can be used as feature representations for classification tasks [17], and we show how this feature is discriminative.

**Figure 5:**
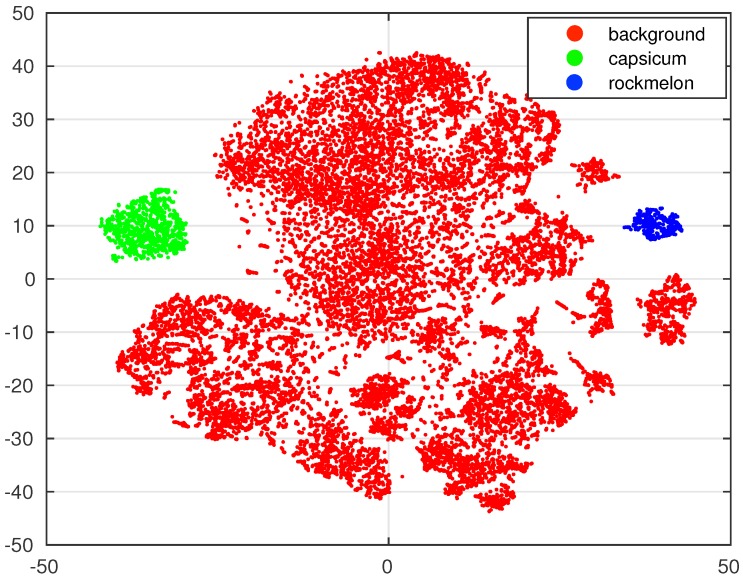
t-SNE feature visualisation of 3 classes. The 4k dimensions of features are extracted from the Fc7 layer and visualised in 2D. For the visualisation, 86 images are randomly selected from the dataset and processed for the network shown in Figure 3.

Four thousands ninety six dimensions of feature vectors are extracted from the fully-connected 7 (fc7) layer and are fed into t-Distributed Stochastic Neighbour Embedding algorithm (t-SNE) [32] with the corresponding labels. t-SNE is one of the popular dimensionality reduction methods that measures pairwise neighbouring similarities using the L2 norm distance in both high and low dimensions. The pairwise similarities are calculated around the sample points, and the Kullback–Leibler divergence is used to gauge the distance between two probability distributions (i.e., the similarities of high and low dimensions). Stochastic Gradient Decent (SGD) minimises the distance to keep the local structure in a low dimension space. Figure 5 shows low dimension (2D) feature visualisation using t-SNE. Each point represents a feature, and its colour is the corresponding label. It is obvious that sweet peppers (green) and rock melons (blue) are highly distinguishable from each other and the background (in red). This figure also shows that good detection results are expected given a reasonable classifier.

Note that the key contributions of this model (VGG-16) are in demonstrating that the depth of the network plays significant roles for proper detection performance, and despite its slightly inferior classification power, its features generated from the network architectures outperform other state-of-the art networks, such as AlexNet [17], ZF [33] and GoogLeNet [34]. It is, therefore, the most popular choice at the time of writing this article in the computer vision and machine learning communities for the front-end feature extraction module. Faster R-CNN also makes use of these feature maps as the guidance for where to look. We will present how to train VGG-16 net and deploy it for fruits detection in the following section.

### 3.3. DeepFruits Training and its Deployment

The data that we have are multi-modal, colour (RGB) and NIR in nature, and so, we fine-tune (adapt) the Faster R-CNN for each modality independently. Fine-tuning consists of updating, or adapting, the model parameters using the new data. In practice, this involves initialising a new classification layer and updating all of the layers, for both the region proposal and classification network. The classification network uses the same architecture as VGG [30], as this provided the best performance.

The VGG network configuration used (Configuration D) consists of 13 convolutional layers followed by two fully-connected layers, referred to as VGG-D. The original implementation of Faster R-CNN was fine-tuned using the PASCAL VOC dataset (20 objects, 11 k images and 27 k annotated objects), and the network was initialised by the pre-trained ImageNet dataset, which consists of 1000 object categories, 1.2 million images and their bounding box annotations [5]. This implies that we are required to fine-tune again the network using our custom data; otherwise, Faster R-CNN can only detect the 20 ordinary objects on which the network was trained, such as aeroplane, bicycle, bird, cat, dog, and so on. By doing this, we can make use of features learned from a large-scale dataset which are well generalised to various visual recognition tasks.

Given the VGG-16 network, we define three classes named ‘background’, ‘sweet pepper’ and ‘rock melon’ and fine tune the network. Regarding this fine-tuning topic [35], abundant resources are available from online [36], and we also have made publicly available our implementation and tutorial document [6].

Table 1 shows the number of training images used by CRF and Faster R-CNN only for the performance evaluation. We can only use a relatively small number of images due to the limited pixel-wise image annotation datasets from [4]. For a fair comparison, the same training and testing images are utilised, and the experimental results are presented in Section 4.2. We also conduct further experiments by increasing the number of classes and training images to detect another fruit and to demonstrate its generalisation.

After the training, we deploy the trained fruit detector on a laptop that has Intel i7, 64-bit 2.90GHz quad-core CPUs, a GeForce GTX 980M 8GB GPU (1536 CUDA cores) and 16GB of memory space running on an Ubuntu 14.04 Linux system. Input images are obtained from a multi-spectral camera, the JAI AD-130GE, and a Microsoft Kinect 2. Each camera has a resolution of 1296 × 964 and 1920 × 1080, respectively. Processing for the detection takes an average of 341ms with a 4ms standard deviation for JAI and 393ms with 3ms for the Kinect 2 image. The processing time gap is caused by an external library for reading different resolution images.

### 3.4. Multi-Modal Fusion

In the previous section, we introduced the proposed fruit detection approach using the Faster R-CNN framework; here, we present the two methods, late and early fusion, that we use to combine the multi-modal (RGB and NIR) imagery that we have. Late fusion combines the classification decisions from the two modalities. Early fusion alters the structure of the input layer of the VGG network so that 4 channels, rather than 3, are provided.

#### 3.4.1. Late Fusion

Late fusion combines the classification information from the two modalities, colour and NIR imagery. Using the independently-trained models for each modality (see Section 3.2), we combine the classification information in the following manner.

Each modality *m* produces $N_{m,P}$ region proposals. To combine the two modalities, these region proposals are combined to form a single set of $N_{P*}=m\times N_{m,P}$ region proposals. A score $s_{m,p}$ is then proposed for the *p*-th proposed region of the *m*-th modality. A single score for the *p*-th region is produced by averaging the response across the modalities,

(1) $$s_{p}=\sum_{m=1}^{M}s_{m,p}$$

The score is a *C*-dimensional variable, where *C* is the number of classes to be classified.

#### 3.4.2. Early Fusion

**Figure 6:**
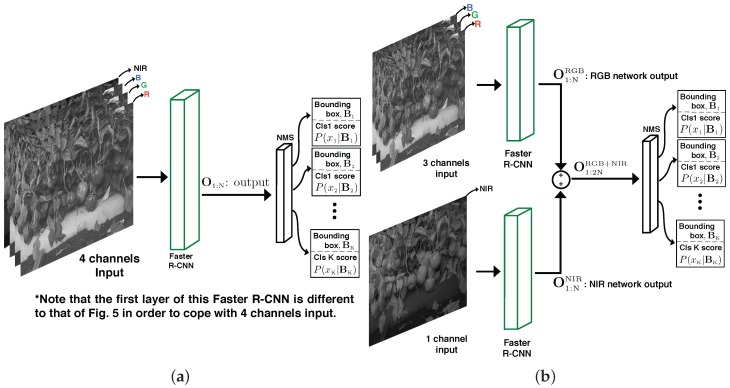
A diagram of the early and late fusion networks. (**a**) The early fusion that concatenates a 1-channel NIR image with a 3-channel RGB image; (**b**) The late fusion that stacks outputs, $O_{1:2N}^{\mathrm{RGB}+\mathrm{NIR}}$, from two Faster R-CNN networks. $O_{1:N}^{\mathrm{RGB}}$ and $O_{1:N}^{\mathrm{NIR}}$ represent the output containing N=300 bounding boxes and their scores from the RGB and NIR networks, respectively. *K* is the number of objects being detected. Note that the Faster R-CNNs of the early fusion are identical to that of Figure 3.

Early fusion alters the structure of the input layer of the VGG network so that the input data layer has $N_{c}=4$ channels (3 channels from RGB and 1 channel from NIR), rather than $N_{c}=3$. The VGG network is modified and adapted to receive RGB and NIR information simultaneously. An overview of this is provided in Figure 6. To achieve this, we duplicate the R response from the VGG-D network and initialise the extra, NIR channel using this; the R channel (620–750nm) is chosen, as it is closest to the NIR channel’s wavelength (750–1400nm). This early fusion network is then fine-tuned as previously described.

## 4. Experimental Results

In this section, we qualitatively and quantitatively evaluate our proposed method on five experimental settings: (1) we compare the early and late fusion performance; (2) we evaluate the performance between the baseline algorithm (CRF) and the proposed method; (3) we inspect the performance of RPN; (4) we exam the generalisation of the proposed method by performing spatial-temporal independent condition experiments; (5) we evaluate the extensibility of the proposed approach by applying it to several other fruits.

**Figure 7:**
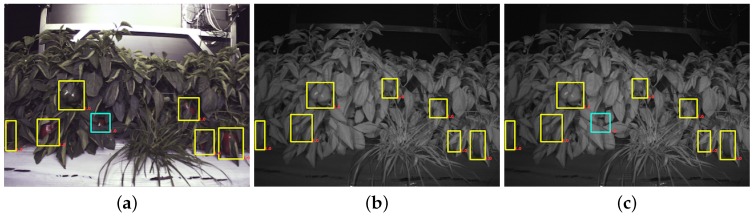
(**a**,**b**) The hand-labelled ground truth using an RGB image and an NIR image respectively; (**c**) A merged ground truth bounding box. The cyan box displays a bounding box that is correctly annotated using the RGB image, but missed in the NIR image, due to the poor visibility of a fruit.

Prior to presenting the experimental results, we mention the creation of the ground truth of the dataset. Figure 7a depicts hand-labelled bounding boxes (yellow) based on the colour image and the NIR image. In Figure 7b, the cyan colour box missing from Figure 7a highlights the missing annotation of a sweet pepper in the NIR image due to its poor visibility; whereas it is more obvious to see the sweet pepper in the RGB image because of the reflection from the sweet pepper. This also happens the other way around. A fruit in the dark is difficult to see in the RGB-based image, but can be identified easily in an NIR image. We thus merge these two ground truth sources using both RGB and NIR images by computing the pairwise Intersection of Union (IoU) of bounding boxes shown in Figure 7c. The remainder of this article refers the merged ground truth as merged GT, and the other two ground truths are referred to as RGB GT and NIR GT based on the image sources used for making the ground truth.

In this paper, we utilise the precision-recall curve with the corresponding F1 score as the evaluation metric for fruit detection. It is considered as detected if the IoU between the prediction and ground truth bounding boxes is greater than 0.4, following [5]. It is worth noting that we choose this threshold as smaller than the ImageNet challenge (0.5) due to the relatively small fruit size with respect to the image resolution. Although the threshold affects the performance evaluations (the smaller the threshold is, the higher the F1 score produced), we consistently use the identical threshold for all experiments and comparisons presented in this paper.

Given this threshold, the precision (P), recall (R) and F1 score are computed as:

(2) $$\begin{array}{c} P=\frac{T_{P}}{T_{P}+F_{P}},\;\;R=\frac{T_{P}}{T_{P}+F_{N}},\;\;F1=\frac{2\cdot P\cdot R}{P+R} \end{array}$$

where $T_{P}$ is the number of true positives (correct detections), $F_{P}$ is the number of false positives (false detection), $F_{N}$ is the number of false negatives (miss) and $T_{N}$ is the number of true negatives (correct rejection).

### 4.1. Early and Late Fusion Performance Comparison

Multi-modal visual sensing techniques are widely used in the agricultural field because they can often capture necessary signatures utilised for detection. We present the results of our proposed early and late fusion methods introduced in Section 3.4. The specifications of the training and testing dataset are shown in Table 1.

**Figure 8:**
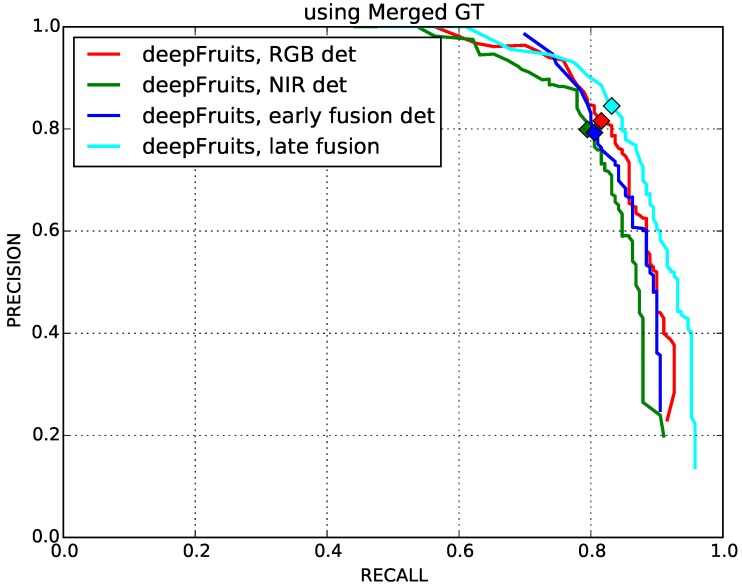
Precision-recall curves of four networks. The marks indicate the point where precision and recall are identical, and F1 scores are computed at these points.

Figure 8 shows the precision-recall curves of single-stream and multi-stream fusion networks. The markers denote the points where precision and recall are identical, and we compute F1 scores at these points shown in Table 2. As expected, the late fusion method outperforms best. It is noted that the late fusion approach contains twice as many parameters (i.e., 276million) than the others (i.e., 138million) and requires more resources, such as computation time and GPU memory space. Interestingly, the RGB network slightly outperforms both the early fusion and NIR network. This may indicate that pre-trained ImageNet parameters are more suitable to operate with RGB inputs. We can also observe that the number of parameters of a network is a critical component to increase detection performance, as mentioned by [25,30].

### 4.2. Fruit Detection Performance Comparison with CRF and Faster R-CNN

As previously mentioned in Section 3.1, fruit detection performance evaluation is conducted between CRF and the fine-tuned Faster R-CNN. We use the same training and test settings as described in Table 1. The only difference is that the pixel-annotated training set is utilised as the ground truth for CRF training, while bounding box annotations are used for Faster R-CNN (see Figure 2). The ground truth for test images remains identical. We should note that the output from CRF is a pixel-level likelihood map representing how much the pixel belongs to a specific label. In order to have a fair comparison with the bounding box outputs of Faster R-CNN, we use a Laplacian of Gaussian (LoG) multi-scale blob detector [37] for the CRF-based method to produce detected fruit regions (i.e., bounding boxes).

**Figure 9:**
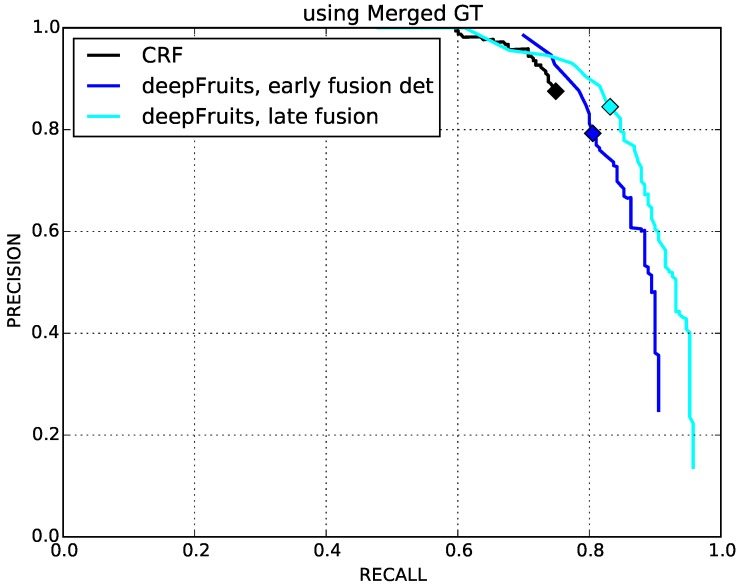
Precision-recall curves of the CRF baseline, early and late fusion networks. All make use of RGB and NIR images as inputs. Due to the performance issue of CRF, we calculate the F1 score slightly offset from the equilibrium point.

Figure 9 shows the precision-recall curves for the CRF and fused networks. CRF has a similar performance as early fusion, but could not reach late fusion’s performance (shown in Table 3). Note that we can only compute the F1 score of CRF from the valid point where precision and recall are slightly different (see the black markers from Figure 9) due to the denominators of precision and recall from Equation (2) being all zeros. This implies that there are no sweet peppers in the ground truth ($T_{P}=0$); therefore, no false detection ($F_{P}=0$) or misdetections ($F_{N}=0$) are reported.

Although CRF shows impressive performance, there are a couple of challenges; difficulty in pixel-level ground truthing and huge processing time. For example, the processing time in order to produce the results shown in Figure 9 takes 331s/frame with a 17s standard deviation for featurisation, which extracts and prepares features for subsequent detection, and 0.819s with a 0.067s standard deviation for the detection; while Faster R-CNN can run of 393ms/frame including all procedures (842-times faster than CRF).

Unfortunately, we are unable to measure the time spent on pixel-level and bounding box annotation, because it is highly subjective with the human-in-loop and the institutes’ internal ethics and integrity issues. However, from empirical experience, doing the bounding box annotation is much faster than pixel-level annotation.

### 4.3. Inspection of RPN Performance

High-quality region proposals significantly contribute to the performance of the object detection task. In this section, we present a study to evaluate proposal quality by demonstrating the detection rate versus a number of proposals being generated, as well as efficiency in terms of region proposal generating time.

Given the sweet peppers in the ground truth, we compute a pairwise IoU for all proposals. If the IoU is greater than a threshold, 0.4, then we consider it as being detected ($T_{P}$), otherwise as missed detection ($F_{N}$). The detection rate (recall) is calculated using Equation (2) with varying numbers of proposals (six levels).

**Figure 10:**
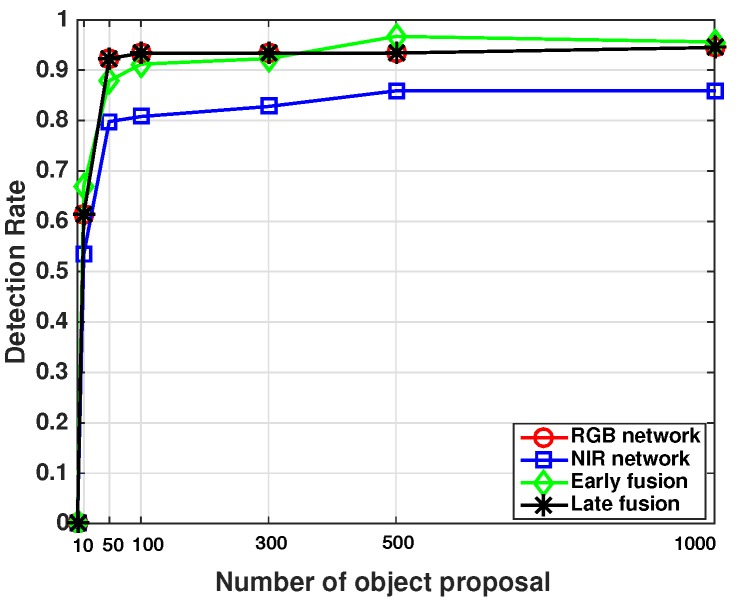
Performance evaluation of the region proposals of four different networks.

It can be observed in Figure 10 that all networks’ performances are almost saturated after generating 100 proposals. There is a subtle improvement at 500 for the NIR network and the early fusion network, but a longer computation time is required. The maximum detection rates are 0.945, 0.859, 0.967 and 0.945 for RGB, NIR, early fusion and late fusion networks, respectively. This implies that the maximum performance that we can achieve is bounded to these maximum detection rates. RGB and late fusion networks report identical performance, since the latter one simply concatenates proposals from both the RGB and NIR networks

This study also explains the incomplete curves in Figure 8. The maximum performance all networks can achieve is less than one due to the limited performance of RPN. For CRF, it can only achieve 0.75 of the maximum detection rate.

The proposals’ generation time demonstrates the efficiency of this method; the results are presented in Table 4. It can be seen that the computation time increases almost proportionally as the number of proposals increases.

### 4.4. Impact of the Number of Training Images for Fruit Detection

We presented the impact of the number of proposals versus the fruit detection rate in the previous section. In this section, we address the study of the impact of the number of images being used for retraining the network. The aim of this study is to demonstrate the performance of the fruit detector by varying the number of training image sets.

**Figure 11:**
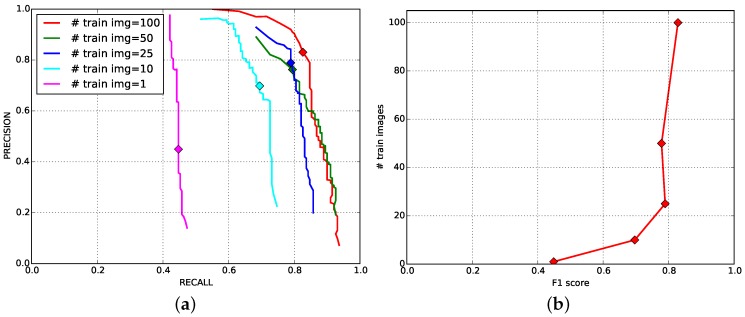
(**a**) Precision-recall curves with the varying number of training images as denoted by different colours. The marks indicate the points where precision and recall are identical; (**b**) The F1 scores versus the number of images being used for fine-turning.

It is known that more training images leads to better performance under the framework of the deep convolutional neural network. The same trend can be seen in Figure 11. It is, however, interesting that the impact of fine-tuning shows impressive results, as only 10 training images are utilised for retraining, but produce a 0.7 F1 score. Using 50 images (green) yields a slightly lower F1 score than using 25 images (blue), but it coversa wider recall area with higher precision. In addition, this study also implies that it is feasible to achieve better performance with more fruit images.

### 4.5. Spatial-Temporal Independent Condition Experiments

There has been abundant interest in the computer vision and machine learning communities [33,38] to study the generalisation of the learning algorithm, as it is one of the important tasks in object detection. A well-generalised model is expected to perform as well as the performance on the training dataset. Recently, it has turned out that training a deep learning model using the massive ImageNet database generalises well to other datasets. It considerably outperformed many hand-crafted feature representations.

In this experiment, we introduce variations to the camera setup, time and locations of data acquisition. Three different cameras are used; a JAI multi-spectral camera, Kinect 2 (RGB only) and a mobile phone camera. The time for data collection is day and night, and the sites are different commercial farms and grocery stores. For training, 100 sweet pepper samples are collected at a commercial farm site, and 109 rock melon images are gathered from a glass house. Experimental results are only demonstrated qualitatively.

**Figure 12:**
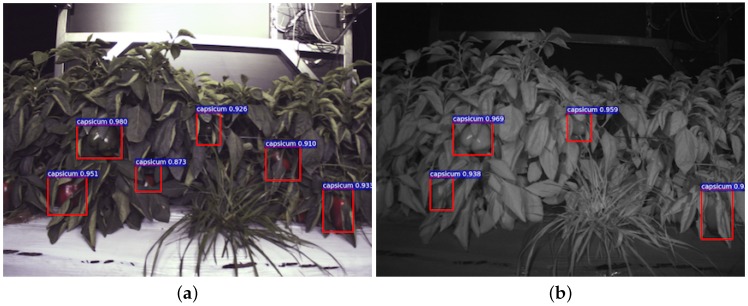
Instances of detection performance using the same camera setup as the training dataset and the same location. Above each detection is the classification confidence output from the DCNN. (**a**,**b**) The outputs from the RGB and NIR networks, respectively. It can be seen that there are noticeable $F_{N}$ (miss) in the NIR image, and colour and surface reflections play important roles in detection for this example.

**Figure 13:**
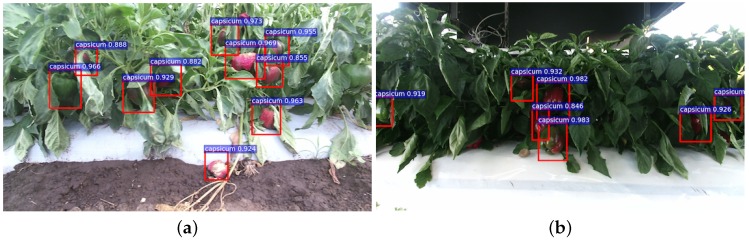
Instances of detection performance using a different camera setup (Kinect 2) and a different location. Above each detection is the classification confidence output from the DCNN. (**a**,**b**) Without/with a Sun screen shed, respectively. Despite the brightness being obviously different in the two scenes, the proposed algorithm impressively generalises well to this dataset.

Firstly, Figure 12 shows sweet pepper detection results for a test image pair (registered RGB and NIR images) that were recorded with the same camera setup (JAI) at the same site at night-time, where the training dataset was collected. There are noticeable $F_{N}$ (missing) in the NIR test image. Figure 13 displays the results of images captured by a different camera (Kinect 2) at a different commercial farm sites during the day time. We can only conduct experiments over RGB images in this experiment, because it is a challenge to register RGB and NIR images obtained from a Kinect 2 due to differences in resolution and visual appearances.

### 4.6. Different Fruit Detection Results

Prior to this section, we have presented only one fruit’s detection performance (i.e., sweet pepper). Detection results for several fruits can be easily obtained through a minor modification of our proposed system; (1) creating a new fruit training dataset (i.e., bounding box annotation for each fruit); (2) performing fine-tuning and deploying the trained model to the new test set. For the steps above, we provide supplementary documents [6]. Note that we trained the network as a one versus rest manner in these experiments. This means that there are only two classes (e.g., ‘background’ and ‘fruit1’). This is acceptable in real applications, because usually, one fruit is cultivated at a one farm site in practice due to economic reasons, such as fertilisation, irrigation and the prevention of harmful diseases and insects.

In this section, we demonstrate the detection of six more novel fruits, including rock melon, strawberry, apple, avocado, mango and orange, with a small training dataset, as shown in Table 5. The datasets for sweet pepper and rock melon are collected by our team, and the rest are obtained from a Google Image search. We choose fruit images in orchard environments. The training and testing dataset will be available upon publication.

For training, we also include different ripeness levels and varieties in order to show the robustness of the detector. A simple colour detector cannot achieve this performance due to the presence of ambiguity in the background. For example, the apple detection test set consists of red and green, and the strawberry test set encapsulates well-ripened red and whitish and greenish young strawberries.

**Figure 14:**
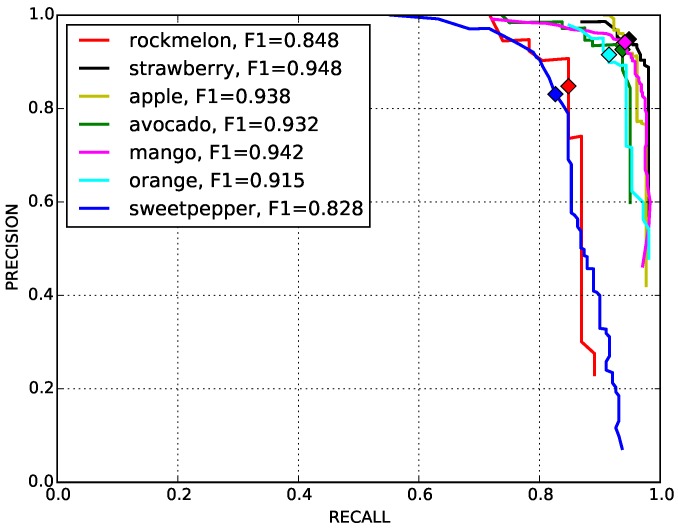
Quantitative performance evaluation for different fruits. The marks indicate the point where F1 scores are computed.

**Figure 15:**
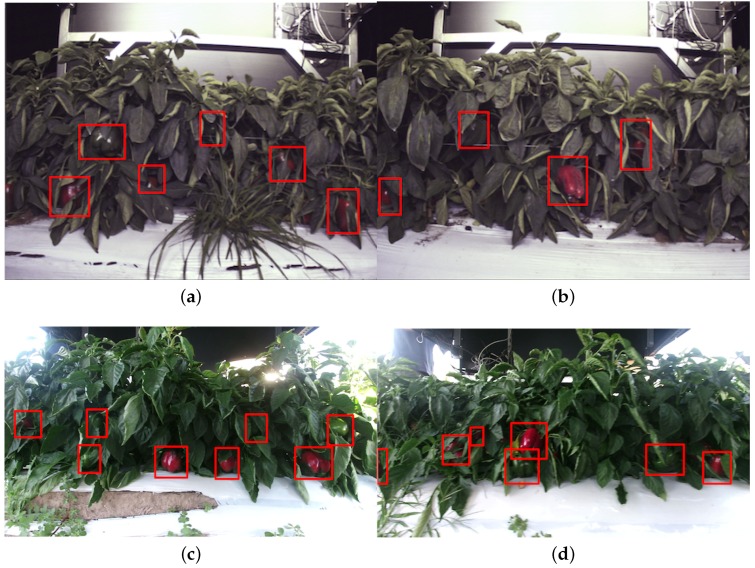
Four instances of sweet pepper detection. (**a**) and (**b**) are obtained from a farm site using a JAI camera, and (**c**) and (**d**) are collected using a Kinect 2 camera at a different farm site. Above each detection is the classification confidence output from the DCNN.

**Figure 16:**
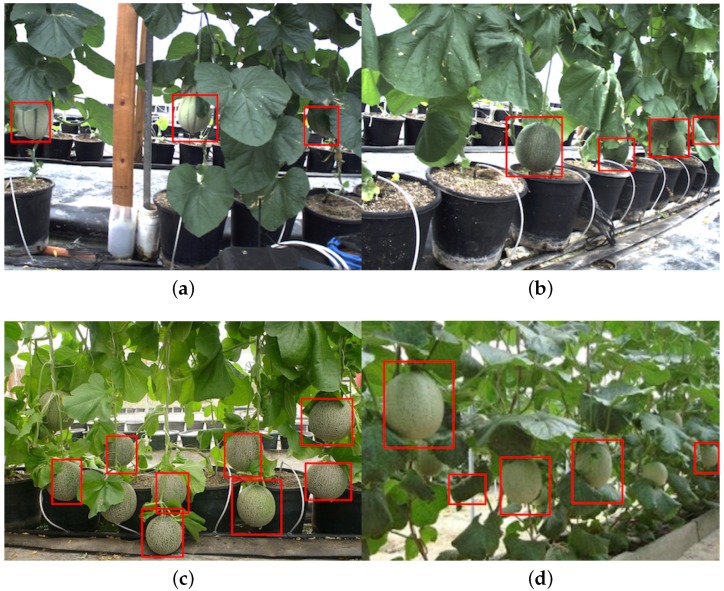
Four instances of rock melon detection. (**a**) and (**b**) are obtained from a farm site using a JAI camera, and (**c**) and (**d**) are from Google Images. Above each detection is the classification confidence output from the DCNN.

**Figure 17:**
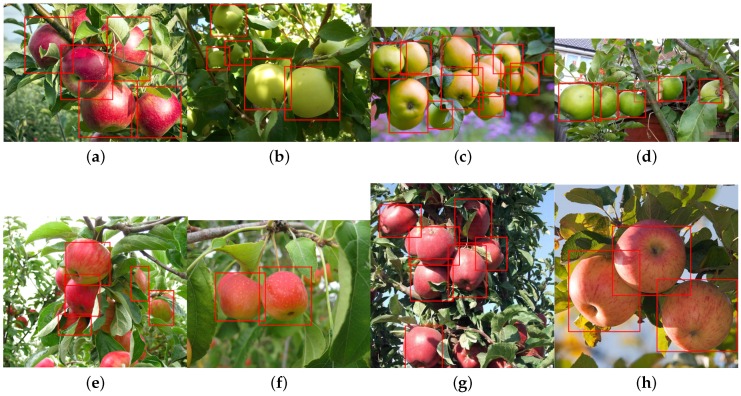
Eight instances of red (**a**,**e**–**h**) and green (**b**–**d**) apples detection (different varieties). Images are obtained from Google Images. Above each detection is the classification confidence output from the DCNN.

**Figure 18:**
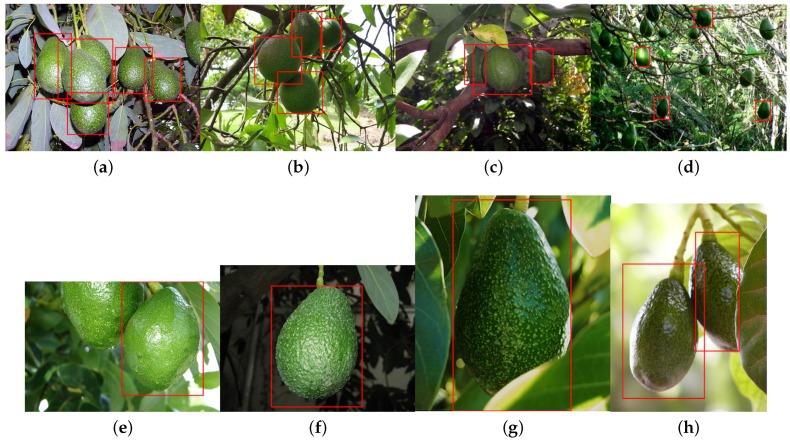
Eight instances (**a**–**g**), and (**h**) of avocado detection (varying levels of ripeness). Images are obtained from Google Images.

**Figure 19:**
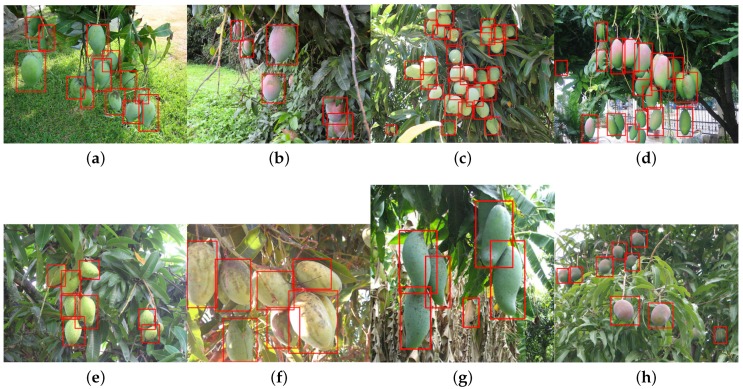
Eight instances (**a**–**g**), and (**h**) of mango detection (varying levels of ripeness). Images are obtained from Google Images.

**Figure 20:**
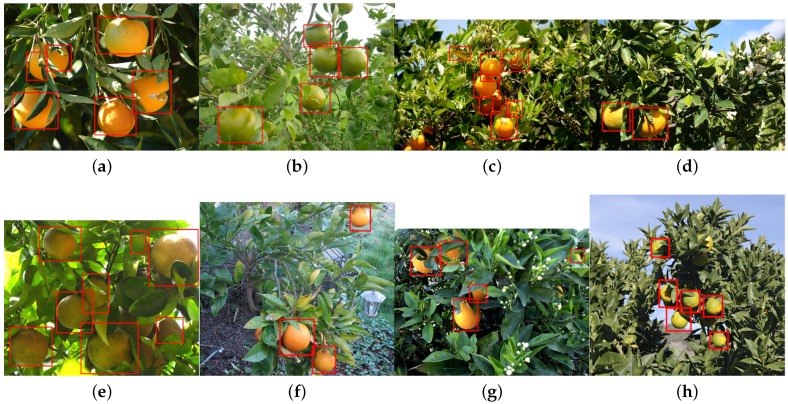
Eight instances (**a**–**g**), and (**h**) of orange detection (varying levels of ripeness). Images are obtained from Google Images.

**Figure 21:**
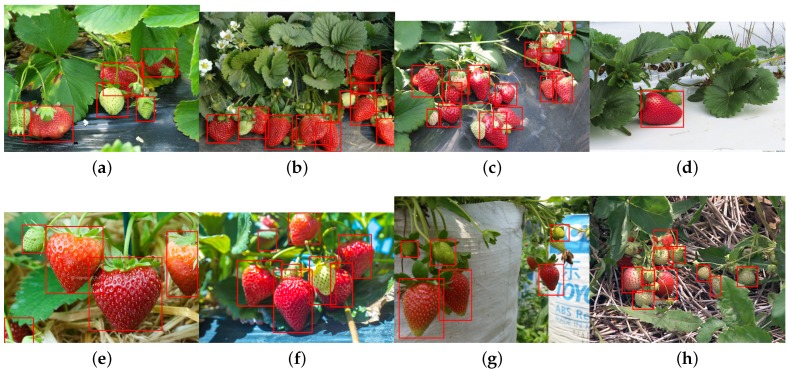
Eight instances (**a**–**g**), and (**h**) of strawberry detection (varying levels of ripeness). Images are obtained from Google Images.

Figure 14 shows quantitative results for seven fruits as precision-recall and F1 scores. We achieve promising results for all fruits. Although the number of the testing dataset is small due to limited accessible labour for data annotation, F1 scores are all above 0.8. It can be observed that the detection results of sweet pepper and rock melon are slightly inferior compared to other fruits. This is mainly due to the large occlusions of fruits at farm sites, as shown in Figure 15 and Figure 16. For qualitative inspection, we present eight instances of fruit detection, except sweet pepper and rock melon (low visuality due to small fruits) throughout Figure 17, Figure 18, Figure 19, Figure 20 and Figure 21.

**Figure 22:**
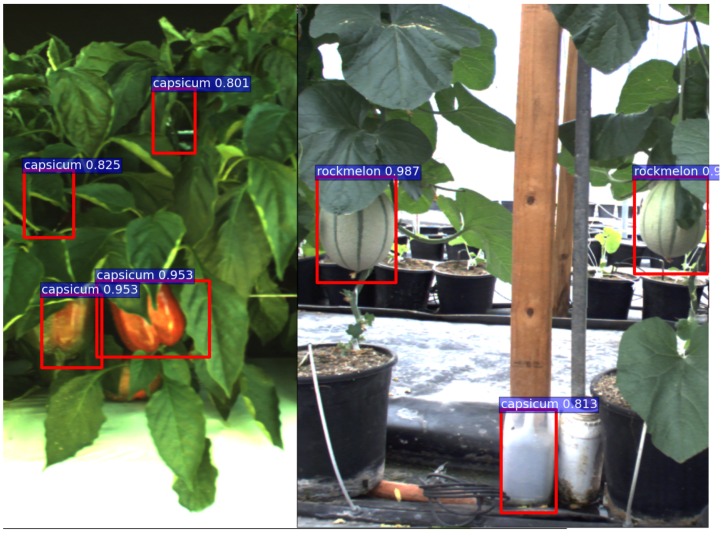
Detection result when two fruits are present in the scene. Two images are manually cropped, stitched and then fed to the RGB network.

In the last experiment, we evaluate our detector to detect several fruits in the same scene as depicted in Figure 22 (using a multi-class detector). There are five fruits in total in the scene; three sweet peppers and two rock melons; and $T_{P}=4$ (hit), $F_{P}=3$ (false detection) and $F_{N}=1$ (miss). In this example, precision is 0.57 with a 0.8 recall rate with a score threshold of 0.8. Note that all $F_{P}$ have relatively low scores (i.e., lower than 0.85), whereas fruits being detected are all above 0.9. If the score threshold is set to 0.85, then precision will be 1.0 with a 0.8 recall rate. This score threshold should be properly adjusted depending on the circumstances and applications.

## 5. Discussion and Future Works

In this section, we discuss the fruit detection results presented in the previous section. It is noticeable that there are several $F_{N}$, false negatives (miss), in Figure 18. The cause can be from two possibilities: the small set of training images; and the scales of fruits in the testing images can be considerably different. We perform a supervised machine learning task that trains a model of the object of interest. A deep neural network can reasonably handle some variations in scales due to its deep convolutional layers. More specifically, the VGG net consist of 13 convolutional operations (3 × 3 kernels), five max pooling layers (2 × 2 kernels) and three fully-connected operations. However, if an object size in a testing image is significantly less than that of a training set, it misses the detection. Empirically, we have witnessed that the proposed detector can handle around 50% scaled-down object detection.

In order to overcome this issue, high-quality and more training image sets covering multiple scale levels are required. We only used 43 training images of avocado and 11 for testing as presented in Table 5.

Fruit counting is also one of our future works. This task includes data association, which means we need to distinguish whether a fruit is already seen from the previous image frame or not [11]. Feature matching, tracking and association techniques are required to identify fruits.

In order to deploy the developed system into real robot systems (e.g., unmanned ground vehicles), the only bottleneck is processing performance, which requires an NVIDIA GPU (Graphics Processing Unit) device that has more than 8 GB of memory for model testing.

## 6. Conclusions

We present approaches for a vision-based fruit detection system that can perform up to a 0.83 F1 score with a field farm dataset, maintaining fast detection and a low burden for ground truth annotation. This is a competitive result compared to our previous pixel-based detector of 0.80. We also demonstrated qualitative results to show how well the trained model using a small dataset generalises to entirely independent (unseen) environments.

In developing this system, we performed fine-tuning of the VGG16 network based on the pre-trained ImageNet model. The novel use of RGB and NIR multi-modal information within early and late fusion networks provides improvements over a single DCNN. Furthermore, we investigated the performance of region proposal networks to narrow down a possible bottleneck of performance degradation. Our findings are returned to the relevant communities through an open dataset and tutorial documentation.

Future work involves the integration of the proposed algorithm with our custom-built harvesting robot and the collection of an enormous amount of ground truth annotations for a variety of fruits by utilising Amazon Mechanical Turk or other out-sourcing supplies to achieve more accurate performance.

## Figures and Tables

**Table 1:** Number of images used for training and testing for CRF and Faster R-CNN.

	Train	Test	Total
	(RGB + NIR)	(RGB + NIR)	
CRF and Faster R-CNN	100 (82%)	22 (18%)	122

**Table 2:** F1 scores of fused and single-stream networks.

RGB Only	NIR Only	Early Fusion	Late Fusion
0.816	0.797	0.799	**0.838**

**Table 3:** F1 scores of CRF and fused networks.

CRF	Early Fusion	Late Fusion
0.807	0.799	0.838

**Table 4:** Region proposal generating time including detection.

	# Prop.	10	50	100	300	500	1000
Net.							
RGB network (in s)		0.305	0.315	0.325	0.347	0.367	0.425
Early fusion (in s)		0.263	0.268	0.291	0.309	0.317	0.374

**Table 5:** Number of images used for training and testing for different fruits.

Name of Fruits	Train (# Images), 80%	Test (# Images), 20%	Total, 100%
Sweet pepper	100	22	122
Rock melon	109	26	135
Apple	51	13	64
Avocado	43	11	54
Mango	136	34	170
Orange	45	12	57

## Abbreviations

The following abbreviations are used in this manuscript:

Faster R-CNN: Faster Region-based CNN
DCNN: Deep Convolutional Neural Network
NIR: Near-Infrared
IoU: Intersection of Union
CRF: Conditional Random Field
SAE: Sparse Autoencoder
CART: Classifier and Regression Trees
PASCAL-VOC: PASCAL Visual Object Classes
RPN: Region Proposal Network
LBP: Local Binary Pattern
HoG: Histogram of Gradients
t-SNE: t-Distributed Stochastic Neighbour Embedding
SGD: Stochastic Gradient Decent
ILSVRC: ImageNet Large-Scale Visual Recognition Challenge
GT: Ground Truth
